# Evolution of the immune system in humans from infancy to old age

**DOI:** 10.1098/rspb.2014.3085

**Published:** 2015-12-22

**Authors:** A. Katharina Simon, Georg A. Hollander, Andrew McMichael

**Affiliations:** 1 Nuffield Department of Medicine, Weatherall Institute of Molecular Medicine, University of Oxford, Oxford OX3 9DS, UK; 2 Department of Paediatrics, Weatherall Institute of Molecular Medicine, University of Oxford, Oxford OX3 9DS, UK; 3 Nuffield Department of Medicine Research Building, University of Oxford, Old Road Campus, Oxford OX3 7FZ, UK

**Keywords:** adaptive immunity, innate immunity, infections

## Abstract

This article reviews the development of the immune response through neonatal, infant and adult life, including pregnancy, ending with the decline in old age. A picture emerges of a child born with an immature, innate and adaptive immune system, which matures and acquires memory as he or she grows. It then goes into decline in old age. These changes are considered alongside the risks of different types of infection, autoimmune disease and malignancy.

## 1. Introduction

And one man in his time plays many parts,

His acts being seven ages.

William Shakespeare^1^

**Figure 1.**
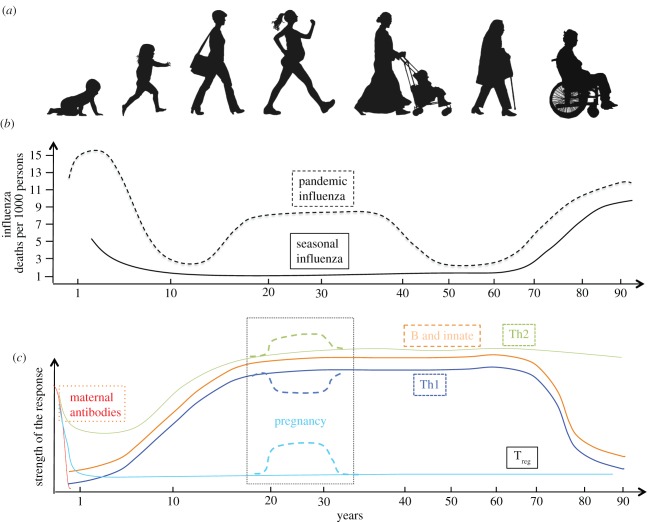
(*a*) The seven ages of woman. (*b*) Schematic graph of excess deaths from seasonal or pandemic influenza over the lifetime of an individual represented as number of deaths per 1000 persons (adapted from [2]). Note that while pregnancy increases the risk of severe influenza, in severe pandemics such as 1918/1919 there were also excess deaths in previously healthy young adults who were not pregnant. (*c*) Schematic graph of the different arms of the immune response to influenza over the lifetime of an individual.

More than 1600 genes are involved in innate and adaptive immune responses [1]. These genes are of great importance for sustaining life in a hostile environment. Yet the immune system is relatively immature at birth and has to evolve during a life of exposure to multiple foreign challenges through childhood, via young and mature adulthood (including pregnancy), to the decline of old age (figure 1).

## 2. Ontogeny of the immune system in early life

   At first the infant,

Mewling and puking in the nurse's arms.

*In utero*, the fetal environment demands that the immune system remains tolerant to maternal alloantigens. After birth, the sudden enormous exposure to environmental antigens, many of them derived from intestinal commensal bacteria, calls for a rapid change to make distinct immune responses appropriate for early life.

### (a) The innate immune system

The innate immune system provides an early first line of defence against invading pathogens. The cells involved are neutrophils, monocytes, macrophages and dendritic cells, which all interact with the adaptive immune system. These cells develop and mature during fetal life, but at different times, and the function of all components of innate immunity is weak in newborns compared with later life.

Mature neutrophils are present at the end of the first trimester and steeply increase in number, stimulated by granulocyte-colony-stimulating factor, shortly before birth. Their number then returns to a stable level within days, but they show weak bactericidal functions, poor responses to inflammatory stimuli, reduced adhesion to endothelial cells and diminished chemotaxis [3]. These deficits are more striking in preterm infants, which also have lower serum IgG and complement. Consequently, the newborn, and especially premature infants, have impaired neutrophil functions [4], putting the child at risk of bacterial infections.

In preterm and newborn infants, classical monocytes and macrophages are also immature. They have reduced TLR4 expression [5] with impaired innate signalling pathways [6–8], resulting in diminished cytokine responses compared with adults. Consequently, there is poor tissue repair, impaired phagocytosis of potential pathogens and poor secretion of bioactive molecules. However, while there is a reduced frequency of pulmonary macrophages in premature and term infants, adult levels of these cells are reached within days after birth [9].

Compared with blood from children or adults, cord blood contains fewer myeloid-type dendritic cells (mDC). They express lower cell surface levels of HLA class II, CD80 and CD86 than adult mDC [10]. They secrete low concentrations of IL-12p70 in response to activating innate stimuli [11]. Thus priming of Th1 and CD8 T-cell responses is diminished compared with adults, correlating with an increased susceptibility to infections caused by viruses, *Mycobacterium tuberculosis* and *Salmonella* spp*.* In contrast, newborn mDC stimulated via TLR4 secrete adult-like concentrations of pro-inflammatory cytokines [12] that promote Th17 immune responses.

Plasmacytoid dendritic cells (pDC) release high concentrations of type I interferon (IFN) in response to TLR7 and TLR9 stimulation in adults. However, newborn pDC are severely limited in secreting interferon α/β upon exposure to different viruses, despite expressing levels of TLR7 and TLR9 that are similar to adults [13]. Consequently, innate immune responses to viruses such as respiratory syncytial virus, herpes simplex virus and cytomegalovirus are poor compared with later in life.

Natural killer (NK) cells in adults restrain viral replication and dissemination before adaptive immunity is established [14]. They are regulated by inhibitory receptors that recognize HLA-A, B, C and E, and therefore contribute to self-tolerance. In early gestation, NK cells are hypo-responsive to target cells lacking major histocompatibility complex (MHC) class I molecules (such as trophoblast [15]) and are highly susceptible to immune suppression by transforming growth factor-β (TGF-β). NK cytolytic function increases during gestation but is still only half of adult level at birth. Neonatal NK cells are less responsive to activation by IL-2 and IL-15, and produce limited IFN-γ concentrations. However, the cells' threshold for activation is lower, which provides some anti-viral protection [15].

The three independent pathways that activate the complement system are critical to host defence and inflammation. Complement components facilitate opsonization, are chemo-attractants for innate cells, mediate cell lysis and influence antibody production. Newborn serum concentrations of almost all circulating components are 10–80% lower than in adults [16], with diminished biological activity. Complement levels increase after birth, with some serum factors reaching adult concentration within a month (e.g. Factor B), but others evolve more slowly [16]. Because infants have low immunoglobulin concentrations, complement effector functions depend on the alternative and lectin-binding activation pathways, triggered by polysaccharides and endotoxins.

Overall, the innate immune system is muted at birth, a price probably paid by the fetus not only to tolerate non-shared maternal antigens but also to ignore the considerable amount of stress and remodelling that takes place during development. This makes the newborn, and particularly the premature baby, relatively susceptible to bacterial and viral infections.

### (b) The adaptive immune system

T cells develop in the thymus, which is largest at birth and during the first years of life. Mature single CD4^+^ and CD8^+^ positive T cells are first detected in the thymus at week 15 and abundant in the periphery well before birth [17,18]. However, neonatal T cells differ significantly from adult cells, reflecting the fetal life, where exposure to foreign antigens is largely restricted to non-inherited maternal alloantigens. The function of early-life T cells is different from adult T cells. For example, though fetal naive CD4^+^ T cells respond strongly to alloantigens, they tend to develop towards Foxp3^+^ CD25^+^ regulatory T cells (T_reg_) through the influence of TGF-β [19], and thus actively promote self-tolerance. Peripheral T_reg_ represent around 3% of total CD4^+^ T cells at birth [20] and these cells persist for an extended period of time [21], giving the early-life immune response an anti-inflammatory profile [22].

Foreign antigen activation of late fetal or neonatal T cells results in a response skewed towards Th2 immunity [23], which is reinforced by neonatal dendritic cells and epigenetic features [24,25]. Very early-life adaptive T-cell immunity is thus characterized by tolerogeneic reactivity, reduced allo-antigen recognition and poor responses to foreign antigens.

In the newborn, in addition to conventional T cells that recognize peptide antigens in the context of classical MHC molecules, there are populations of γδ T-cell receptor (TCR)-positive and innate-like αβ TCR-positive T cells. These include functionally competent iNKT cells that rapidly produce IFN, mucosal-associated invariant T (MAIT) cells [26] and the newly described interleukin-8 (CXCL8)-secreting naive T cells that bridge innate and adaptive immunity [27]. MAIT cells develop in the thymus, but their maturation can take place in fetal mucosal tissues before microbial colonization. The CXCL8-producing T cells produce important effector functions in human newborns as they have the potential to activate antimicrobial neutrophils and γδ T cells. They appear to be particularly active at the mucosal barriers of premature and term infants, though their frequency decreases with age. In contrast to adult blood, where the repertoire of γδ TCR is restricted, neonatal blood γδ T cells display a variety of receptor chain combinations that change with gestation [27]. γδ T cells can produce significant amounts of IFN-γ, after brief polyclonal stimulation, compensating for the immaturity of the more classical Th1-type T-cell response to neonatal infections [28,29].

Two types of B cell arise via distinct developmental pathways [30]. B1 cells spontaneously secrete low-affinity IgM with a limited range of antigen specificities (including common bacterial polysaccharides), have fewer somatic mutations and serve as a first line of defence [31]. B1 cells secrete IL-10 and TGF-β, and thus promote a Th2 response. At birth, B1 cells comprise 40% of peripheral blood B cells and this frequency remains high for a few months [32]. Conventional B cells (designated B2 cells) originate from a multi-linage CD34^+^ common lymphoid progenitor and generate a broad repertoire of immunoglobulin specificities due to their expression of terminal deoxynucleotidyl transferase, which enhances diversity in V-D-J immunoglobulin gene segment joining. B cells are typically present in secondary lymphoid organs and in the bone marrow, where they contribute to the humoral response of the adaptive immune system.

Most antibody responses, including those to bacterial proteins, bacterial polysaccharides and to polysaccharide–protein conjugate vaccines, are dependent on T-cell help. They rely on interactions between the TCR and the engagement of co-receptors including CD28 and CD40 ligand on Th2 or follicular T helper cells with their corresponding binding partners HLA-peptide, CD80/86 and CD40 on antigen-specific B cells. However, neonatal B cells express low levels of these co-receptors, limiting their capacity to respond [33]. Furthermore, low levels of the receptor for complement C3d fragment (CD21) impede responses to polysaccharide–complement complexes [34]. Together, these features contribute to blunted humoral immune responses with incomplete immunoglobulin class switching [35], although memory B cells are generated [36]. B cells from neonates and infants aged less than 2 months show decreased somatic hypermutation compared with adults, limiting affinity maturation of antibodies [37]. Finally, there is a failure of early-life bone marrow stromal cells to support long-term plasmablast survival and differentiation to plasma cells, so that any IgG antibodies elicited rapidly decline after immunization, unlike in older children and adults [38]. Hence, the efficiency of the adaptive immune system to respond to T-cell-dependent antigens early is markedly impaired in neonates compared with older children and adults. This physiological behaviour is particularly relevant to vaccination programmes. Together with the impaired innate immunity, the weak Th1 and antibody responses amply explain why neonatal mortality can be high under conditions of increased pathogen exposure.

## 3. From childhood to adulthood

Then, the whining schoolboy with his satchel

And shining morning face, creeping like snail

Unwillingly to school.

The young human child, even as the innate and adaptive immune systems start to mature, is at risk from many pathogenic viruses, bacteria, fungi and parasites. Nevertheless, he or she has a good chance of survival in developed countries. Before there was good nutrition, hygiene and comprehensive vaccination, there was a high mortality in infants and young children. In 1900, the UK infant mortality rate was 140 per 1000, falling to 7 per 1000 by 2000 [39]. This reduction in mortality was proportionally greater in infants and children compared with other age groups [40]. Better prevention and control of infections accounts for most of this fall. However, in many countries, infant mortality rates remain above 50 per 1000, giving some indication of the evolutionary pressure that must have selected a working protective immune system. Furthermore, such pressure has selected the extreme genetic polymorphism in the MHC, which through peptide presentation to T cells and NK cells is a key regulator of almost all immune responses.

The immune system gradually matures during infancy. Critical early protection against many infectious diseases previously experienced by the mother is given by the passive IgG antibody transferred from the mother transplacentally and in milk. Once that fades away, young children become more vulnerable to infections, though by then better armed with the maturing innate and adaptive immune systems. The risks are now much reduced by vaccinations, which stimulate protective immune responses in the maturing immune system. Nevertheless, children may still acquire viral, bacterial and parasitic infections that have to be fought off and controlled by immune responses. Besides promoting recovery, such antigen stimulation results in immunological memory [41,42]. Thus, over time, protection provided by the immune response increases, and young adults suffer fewer infections. This accumulation of immunological memory is an evolving feature of the adaptive immune response. The memory persists into old age [41] but then may fade.

Besides frank infections and vaccinations, the newborn is exposed to other antigens. He or she comes from a relatively sterile environment *in utero* and is then rapidly exposed to multiple microbes [43]. The first major exposure to bacteria is during passage through the birth canal, and then as soon as he/she makes oral, skin and respiratory contact with the exterior. From then on, exposure to microorganisms is continuous. Many of the bacteria that colonize the gut and other mucosal sites are essential for healthy life, including digestion of food and acquisition of vital nutrients. They also impact on the development of the immune system [44].

Approximately 20% of all lymphocytes reside in the gut [45], exposed to many possible foreign immunogens. Gut immune cells monitor the boundary with a potentially dangerous source of infections. Gut bacteria influence the development of Th17 cells [46], T_reg_ cells [47] and memory T cells [48–50]. At birth, nearly all T cells carry the CD45RA glycoprotein, typical of naive T cells, which have never encountered foreign antigen. There are also relatively abundant T_regs_ within the CD45RA negative CD4 T cells. During childhood, T_reg_ cell numbers decline, and memory Th1, Th17 and Th2 cells gradually increase to equal the number of naive T cells [51]. Although some of these memory T cells could have been stimulated by infections with specific pathogens and by vaccinations, many may be primed by the microbiome, not only in the gut but also in the respiratory tract and skin. These primed memory T cells may respond to subsequent infections through cross-reactions [48,52,53]. For example, adults who have never been exposed to HIV-1 have memory T cells in their repertoire that react with HIV peptides presented at the cell surface by HLA proteins; these T cells are likely to be reawakened should HIV infection occur [48,50], similarly to other microbes [52]. The cross-reactivity arises from the discrete short (8–15 amino acids) peptides (epitopes) which fit into peptide-binding grooves on the HLA class I or II molecules at the cell surface and are then recognized by T cells. Within the microbiome sequences, there are numerous perfect and near-perfect matches to known virus peptide epitopes, such as those from HIV-1 [48,50]. These could easily be responsible for generating the memory T cells specific for pathogen epitopes the person has never encountered.

Segmented filamentous bacteria in the gut are necessary for the development of Th17 cells [47] and *Clostridium* spp. induce colonic T_reg_ cells [54,55]. Germ-free mice have immunological defects, including fewer Peyers patches, smaller lymphoid follicles and abnormal germinal centres in the small intestine lymphoid tissue [56]. This immuno-deficiency can be corrected in a few days by adding a single mouse with normal gut flora to a cage of germ-free animals [56,57]. Thus animal data support the notion that the microbiome shapes the development of both memory T and B cells.

Similar events occur for B cells. The carbohydrate antigens of the ABO blood groups cross-react with gut bacterial antigens and stimulate IgM antibody responses. Antibodies to the gp41 protein of HIV-1 may be derived from B cells whose antibody receptors cross-react with a protein in *Escherichia coli* [58].

As the child grows, the immune repertoire is also shaped by intercurrent infections and vaccinations [59]. Pathogenic infections can be documented by symptomatic illnesses suffered by the child or adult, but for many viruses, such as influenza, infection may be subclinical, but still sufficient to stimulate or boost immune responses [60]. Generally, the protection offered by the immune response, both by antibodies and T cells, is very potent. Most childhood infections happen only once and then protection is lifelong.

The maintenance of long-term B-cell memory is remarkable given that IgG immunoglobulin has a half-life *in vivo* of around 25 days [61]. The antibody-producing plasma cells that develop during an immune response migrate to the bone marrow, where they are very long lived. In addition, there may be continuous regeneration of memory B cells in contact with persisting antigen and helper T cells. Particulate antigens persist for years in lymph nodes, held by follicular dendritic cells [62]. Antigen persistence and cross-reactive antigens probably help to keep these B cells alive, dividing occasionally and secreting antibodies.

It is remarkable that a mother can transfer sufficient antibody to protect her infant when she was infected 20–30 years previously. The transmission of protective antibody protection from a mother to her child is hugely important, especially in environments where 15% or more infants and children die of infection. Paradoxically, a mother who avoided a dangerous childhood infection, through herd immunity, may actually put her child at risk by being unable to transfer specific protective antibodies.

There are a large number of asymptomatic chronic infections, mostly viral, that provoke immune responses. Examples are cytomegalo virus (CMV), Epstein–Barr virus (EBV) and *Mycobacterium tubercolosis* (*Mtb*), but the full list is long and expanding [63]. EBV, CMV and *Mtb* provoke very strong CD4 and CD8 T-cell responses in humans. The CMV-specific CD8 T-cell response can result in oligoclonal T-cell expansions reaching more than 10% of circulating CD8 T cells. These T cells are important because they control the virus and their depletion, for instance by immunosuppressive therapy, can activate the infection (e.g. *Mtb*, EBV, CMV), with devastating consequences.

The evolution of antibody responses in B lymphocytes has been reviewed elsewhere in detail [64]. In brief, naive B cells with antibody receptors specific for the immunogen bind antigen in the germinal centre of lymph nodes and receive a partial signal. The bound antigen is internalized and digested in lysosomes. A few resulting peptides bind to the HLA class II molecules of that cell and are then presented on the cell surface where T follicular helper cells with appropriate T-cell receptors respond and deliver further signals, including IL-21, to the B cell. These signals trigger B-cell division, class switching of the antibody genes and somatic hypermutation. B cells that express mutated antibody that binds immunogen with higher affinity are then favoured. Selection for better binding antibodies continues over months, ultimately resulting in high-affinity antibody coming from highly mutated germ line genes. High-affinity antibodies are more effective at neutralizing or opsonizing invading microbes and their pathogenic products.

The somatic hypermutation process does not occur in T cells, even though they have antibody-like T-cell receptor genes, because there is no advantage in having a high-affinity T-cell receptor. The T-cell receptor binding to the peptide–HLA complex on an antigen presenting cells has low affinity. It is enhanced by several co-receptor–ligand pairs that are not antigen-specific, giving the T cell the signal to divide and function.

As a result of an immune challenge, the responding T and B cells may expand transiently to very high numbers [65], sometimes more than 10% of all circulating T cells, but these decline rapidly as a result of activation-induced cell death and from attrition over a longer time period. Thus as the pathogen is controlled and disappears, some memory T and B cells persist for a long time in numbers that far exceed the number of naive and ‘naive-memory’ T cells that were there before infection.

As the individual gets older, he or she develops an expanding repertoire comprising memory T and B cells triggered by previous infections and vaccinations, but also a naive-memory repertoire shaped by exposure to the microbiome, food antigens and inhaled antigens. Given the great complexity of the T- and B-cell repertoires and a large stochastic element in choosing which cells will respond to a given stimulus, and somatic mutations in B cells, the precise composition will differ in each individual, even in monozygotic twins [66]. Add to this considerable genetic variability in how individuals respond, determined by the highly polymorphic HLA genes [67] and by the genes of innate immunity, and it is not surprising that the immune responses of any single adult vary considerably.

### (a) Pregnancy

It is beyond the scope of this review to explore the immunology of pregnancy in detail (reviewed in [68,69]). However, successful reproduction is of central evolutionary importance and there are immunological issues. How the newborn retains mechanisms by which the fetus minimizes its immune responses to the mother has been discussed above. A bigger puzzle is how the mother tolerates a semi-allogeneic graft without rejecting it and without the immunosuppression necessary to accept an organ transplant [70]. There are features at the trophoblast maternal interface at the site of initial implantation and in the placenta that subvert the normal graft rejection immune response. These include expression only of non-polymorphic non-classical HLA antigens on the trophoblast [71], local immune suppression mediated by infiltrating NK cells [72], monocytes and T regulatory cells [69,73], and inhibition of T-cell activation by tryptophan catabolism [74]. Around the time of implantation, a local inflammatory response sets up the stable placental site [68]. There is evidence that the mother changes the balance of her T-cell responses to Th2 rather than Th1 [68]. Thus pregnant women can show remissions of autoimmune disease [75], and are more susceptible to severe complications of influenza [76] and some other infections. This immune modulation, necessary for the well-being of the fetus, can occasionally be harmful to the mother.

### (b) Malignancy and autoimmunity

The primary role of the immune system is probably to protect against infections. Other roles such as destruction of mutated cells may be very important, though more so in old age after reproduction. Many tumours turn off T cells specific for tumour antigens by binding to ‘check-point’ receptors such as PD-1 or CTLA4, and new treatments that block these receptor–ligand interactions have great therapeutic potential [77,78]. However, the side effects of such therapy and of the passive transfer of anti-cancer T cells include autoimmune reactions, suggesting a balance between anti-self-immune reactions preventing cancer and causing autoimmunity [79]. In adult life, the balance usually works, but one-third of Western humans develop cancer, usually later in life, while 5–10% develop clinical autoimmune disease, so the balance is finely set and may shift over time. The fading immune system in old age (see below) may ameliorate autoimmunity but at the expense of increased cancer risk.

Microorganisms cause about a quarter of all cancers (e.g. EBV, hepatitis B and C viruses, human papilloma virus and *Helicobacter pylori*). Specific T-cell responses normally hold these microbes in check. However, if immunity is impaired through ageing (see below), immunosuppressive therapy or certain infections, particularly HIV-1, these cancers emerge [80].

Therefore, having developed a fully effective immune response in early childhood, this matures as memory accumulates and maintains the health of the individual during critical periods of life, including child bearing. It not only protects against potentially lethal infections but also controls a number of persisting infections, some of which have the potential to cause cancer. It can also deal with mutant cells that have potential for becoming malignant. It can be over-reactive and cause autoimmune disease or allergy, a price paid for the overall benefit.

## 4. Immune decline with age

   Last scene of all,

That ends this strange eventful history,

Is second childishness and mere oblivion,

Sans teeth, sans eyes, sans taste, sans everything.

As age advances, the immune system undergoes profound remodelling and decline, with major impact on health and survival [81,82]. This immune senescence predisposes older adults to a higher risk of acute viral and bacterial infections. Moreover, the mortality rates of these infections are three times higher among elderly patients compared with younger adult patients [83]. Infectious diseases are still the fourth most common cause of death among the elderly in the developed world. Furthermore, aberrant immune responses in the aged can exacerbate inflammation, possibly contributing to other scourges of old age: cancer, cardiovascular disease, stroke, Alzheimer's disease and dementia [84].

During a regular influenza season, about 90% of the excess deaths occur in people aged over 65. Furthermore, poor immune responses account for diminished efficacy of vaccines [82,85]. Immune senescence also results in reactivation of latent viruses, such as varicella-zoster virus, causing shingles and chronic neuralgia.

Deterioration of the immune system with age may compromise the homeostatic equilibrium between microbiota and host. Thus reduced bacterial diversity in the gut has been correlated with *Clostridium difficile*-associated diarrhoea, a major complication for the elderly in hospitals [86]. Moreover, deviations from the intestinal microbiota profile, which was established in youth, are associated with inflammatory bowel disease [87]. The increase with age in pro-inflammatory pathobionts and the decrease in immune-modulatory species may promote and sustain inflammatory disorders [86].

At the same time, the ageing immune system fails to maintain full tolerance to self-antigens, with an increased incidence of autoimmune diseases. [88]. This is probably due to lymphopaenia occurring with age, leading to excess homeostatic lymphocyte proliferation [89], as well as a decrease in regulatory T-cell function and decreased clearance of apoptotic cells by macrophages [81].

Cancer is most frequent in older people; the median age for cancer diagnosis in industrialized countries is approaching 70 years of age. The main reason is obviously the accumulation of cellular and genetic damage throughout life; however, given the role of the immune response in controlling cancers, reduced immune functions in the elderly must contribute to the higher risk [90]. This immune impairment is in apparent contradiction to the increase in autoimmunity as anti-tumour responses can be directed against self; however, the general decline of the immune system probably prevails and tumours are no longer rejected as efficiently. Moreover, the increased inflammation found with age facilitates cancer emergence.

The increased morbidity due to the decline of the immune system is a direct consequence of dysregulated adaptive immunity in the elderly. The low number of naive T cells versus T cells [41,42] is a consequence of the reduced thymic output from the involuted thymus. As a consequence of this age-induced lymphopaenia, T cells proliferate and increase the ‘virtual memory’ compartment [91], but at the same time, the ability to establish immunological memory in response to de novo antigens is reduced, compromising vaccinations. Functions such as cytokine production by CD4 and CD8 T cells are impaired, the expression of key surface markers is altered and the CD4^+^ to CD8^+^ T-cell ratio is inverted [81]. The expanded T-cell responses that keep latent viruses such as EBV and CMV under control reduces space for CD8^+^ T cells specific for other potentially lethal viruses [92], exacerbated by the reduced thymic naive T-cell output.

While peripheral B-cell numbers do not decline with age, the composition of this compartment changes. Similar to T cells, naive B cells are replaced by antigen-experienced memory cells, some of which are ‘exhausted’ (CD19^+^IgD^−^ CD27^−^), and they display decreased affinity maturation and isotype switching [81].

In general, the changes in the T- and B-cell compartments hamper the adequate immune response to new acute and latent viral infections and vaccinations.

The innate immune response also declines with age. There are changes in innate cell numbers, with skewing of haematopoiesis towards the myeloid lineages [93,94]. The senescent neutrophil is less functional with decreased phagocytic ability and superoxide production partly due to decreased Fc*γ* receptor expression [95]. Similarly, ageing macrophages have a decreased respiratory burst. Together with DCs, they display reduced phagocytic function and HLA II expression [81]. The immunological ‘silent’ removal of apoptotic and increasing numbers of senescent cells is therefore compromised, and may contribute to the pro-inflammatory phenotype. Indeed, when senescent cells were removed from aged mice artificially, the animals lived longer and were healthier [96].

Possibly the most critical change in the ageing innate immune system is the increase in pro-inflammatory cytokines IL-1β, IL-6, IL-18 and TNFα [97]. The resulting low-grade inflammation probably contributes to atherosclerosis, dementia and cancer, inextricably linking inflammation and ageing of other tissues [84,98].

The cellular and molecular basis of immune senescence is still not well understood. Three phenotypes characterize senescent cells: telomere attrition accompanying each round of proliferation, leading to arrested cell division or ‘replicative senescence’; increased mitochondrial load/dysfunction and reactive oxygen species; and senescence-associated secretory phenotype (SASP), defined as the secretion of pro-inflammatory cytokines, chemokines and proteases by senescent cells [99]. While most of the data have been obtained in fibroblasts, senescent immune cells probably show similar features. These features impact on mitotically active cells by depletion or arrested division (e.g. haematopoietic stem cells—HSCs or T cells), and on post-mitotic immune cells by causing cellular dysfunction (e.g. neutrophils).

Attrition of telomeres is a protective mechanism against cancer, as each round of proliferation is likely to introduce mutations [100]. Only epithelial lymphocytes and stem cells including haemopoietic (HSCs) express the telomere-lengthening enzyme telomerase in the adult [101], requiring a careful balance against the risk of cancer. Both memory T cells and HSCs characteristically divide rarely, to minimize telomere attrition, but reliably either in response to infection (memory lymphocytes) or for tissue renewal (stem cells) throughout the entire lifespan. End-stage senescent CD27^−^CD28^−^ T cells have the shortest telomeres and show decreased proliferation after activation, but nevertheless exhibit potent effector functions. These cells accumulate in old age and in patients with autoimmune diseases and chronic viral infections [102]. The second characteristic of aged cells is increased mitochondrial dysfunction and ensuing oxidative damage to proteins and DNA. DC function in aged mice can be restored through administration of anti-oxidants [103]. Oxidative stress causes DNA breaks and may be the cause of telomere attrition, which links the first two causes of ageing. The accumulation of oxidative damage could be due to a decline in lysosomal and autophagy function [104]. Autophagy, degrading bulk cytoplasmic material by delivering it to the lysosomes, falls with age, including in human CD8^+^ T cells [105]. Mice without autophagy in their haematopoietic system display a prematurely aged haematopoietic system [106]. Failing memory T cells' responses to flu vaccination observed in the elderly can be restored with an autophagy-inducing compound [107]. A third more recent addition to these fundamental changes of aged cells is the acquisition of the SASP, contributing to increased pro-inflammatory cytokine secretion and low-grade inflammation [99].

## 5. Evolution of the human immune system

As a long-lived species, humans have evolved mechanisms of innate immunity and immunological memory to survive recurrent infections. However, over the lifetime of an individual, these immune mechanisms change, first to adapt to the change from fetus to infant, and then to mature and expand during growth, subtly changing in pregnancy and finally decreasing in senescence. The output of naive lymphoid cells and the ability to form new immunological memory becomes increasingly less important as the older individual will have encountered and established a memory bank to many pathogens over its lifetime. There is a possibility that the myeloid bias and the increased secretion of pro-inflammatory cytokines during ageing are essential for improved phagocytosis of an increasing number of senescent cells, raising the question of whether the changes in the ageing immune system might serve a purpose.

The immune system has been primarily moulded by evolution to respond efficiently to acute infections in young people, to adapt to pregnancy and to transmit protection to infants, and is adapted to cope with many chronic infections lasting for decades. Apart from fighting viruses, bacteria, fungi and parasites, the immune system also assumes other roles such as tissue repair, wound healing, elimination of dead and cancer cells, and formation of the healthy gut microbiota. Assuming an absence of a major selective pressure on humans beyond reproductive age, we may have to pay for genetic traits selected to ensure early-life fitness by the later development of immunological phenotypes such as chronic inflammation. Massive ageing and advanced longevity are very recent phenomena occurring in an optimized environment. As proposed by Hayflick [108], ageing may be an artefact of civilization, and hence changes in the ageing immune system might just be a consequence of evolutionary unpredicted antigenic exposure over the lifetime of an individual.

In some aspects, the immune system of the aged organism resembles that of the newborn, with reduced antimicrobial activity by neutrophils and macrophages, reduced antigen presentation by DCs and decreased NK killing, and somewhat compromised adaptive lymphocyte responses. Both the very young and old immune systems are therefore similarly compromised in coping with a typical viral infection such as influenza, whereas the young (non-pregnant) adult organism seems to be perfectly equipped for this challenge (figure 1). The evolution of the immune system within an individual possibly reflects the central role of the young adult in the survival of the species for its procreative potential.

## Endnote

1 All epigraphs in this paper are from William Shakespeare's *As you like it*, act 2, scene 7.
